# Reference curves for refraction in a German cohort of healthy children and adolescents

**DOI:** 10.1371/journal.pone.0230291

**Published:** 2020-03-11

**Authors:** Carolin Truckenbrod, Christof Meigen, Manuela Brandt, Mandy Vogel, Siegfried Wahl, Anne Jurkutat, Wieland Kiess

**Affiliations:** 1 LIFE Leipzig Research Center for Civilization Diseases, University of Leipzig, Leipzig, Germany; 2 Department of Women and Child Health, University Hospital for Children and Adolescents and Center for Pediatric Research, Leipzig University, Leipzig, Germany; 3 Institute for Ophthalmic Research, Eberhard Karls University Tuebingen, Tuebingen, Germany; 4 Carl Zeiss Vision International GmbH, Aalen, Germany; 5 Center for Pediatric Research, Leipzig University, Leipzig, Germany; Keio University School of Medicine, JAPAN

## Abstract

**Purpose:**

Percentile curves of refractive development for German children were generated. We hypothesize that refraction in children in central Europe might differ from data in central Asia.

**Methods:**

Non-cycloplegic refraction was measured using the ZEISS i.Profiler plus (Carl Zeiss Vision GmbH, Germany) in 1999 children, of which were 1046 male and 953 female, aged 3 to 18 years. Reference curves were calculated with the R-package GAMLSS as continuous function of age.

**Results:**

There were only little differences for all centiles between the genders at 3 years and a general trend towards more myopia with increasing age. For the 97^th^ centile and the 3^rd^ centile, girls showed higher myopia/ less hyperopia than boys. Between the age of 3 and 18, the median refraction became -0.68 D and -0.74 D more myopic for boys and girls, respectively. At the same time, the 97^th^ centile for boys changed +0.29 D towards hyperopia and in girls -0.52 D towards myopia. A general myopic trend was seen in the 3^rd^ centile, which was -2.46 D for boys and -2.98 D for girls. For both genders, the median became less than zero at the age of 10 years but did not become myopic (less than -0.5 D) up to the age of 18.

**Conclusion:**

Our analysis presents the first reference curve for refraction in central Europe. In comparison to data from China and Korea, there is only little difference at the age of 5 years in all centiles which then increases continuously. For all ethnicities, a trend towards myopia with increasing age could be observed, but myopia progression is much higher in China and Korea than in Germany. The most marked differences can be seen in the lower centiles. Further investigations should clarify whether commencement of preschool activities with prolonged near-work initiates the divergence in refractive development.

## Introduction

Reference centile curves are commonly used in paediatric practice in order to estimate body height and weight development. They are used as a screening tool to assess the development and well-being of children [1,2].

Chen et al. have introduced the use of reference curves for refractive development in order to identify children at risk for high myopia [3]. Once children at risk to develop high myopia are identified, a treatment to slow down myopia progression can be commenced, like the use of atropine eyedrops, Orthokeratology or contact lenses for myopia treatment [4]. The use of centile curves to predict future refractive development is conclusive, as Zadnik et al. have shown that the current refraction in children is the most important predictive factor for myopia onset [5].

However, centile curves for refraction are population-specific [6,7]. Therefore Chen et al., whose study was carried out in Guangzhou, China, have called for more studies to generate centile curves for refraction [3]. Incidence, progression and prevalence of myopia differs tremendously from Asia to Europe. The prevalence of myopia for children between 5 and 16 years in Hong Kong is 36.7% with a progression from 18.3% at 6 years of age to 61.5% at 12 years of age [8]. In Germany, the prevalence of myopia is 13.3% for the age group 3 to 17 years. The progression of myopia in the age groups 3 to 6 and 11 to 13 was 2.4% to 13.6% and 2.1% to 19.3% for boys and girls, respectively [9]. The trend towards high myopia is much smaller in paediatric cohorts in Germany, compared to Asia [7]. Between 2000 and 2015 the prevalence of myopia did not increase in Germany [10]. High myopia in the adult age is associated with higher risks for complications, such as myopic maculopathy [11] and retinal detachment [12]. But not only children with risk for high myopia can be treated with atropine, orthokeratology or special contact lenses. These options are open to all myopic children [11], although a smaller amount of myopia is associated with a smaller risk for complications [11]. Therefore the use of centile curves is interesting for any population.

Centile curves allow also for comparison of refractive development of different populations. Instead of comparing only the refractive state of the population at a certain age, trends can be compared with reference curves. Finding the age at which refractive development starts to differ between ethnicities may help to find the cause for the difference in myopia prevalence between Asia and Europe. Whereas the differences of myopia among populations are studied thoroughly, there are fewer data about the differences of hyperopia development [13–15]. This gap can be closed by comparison of centile curves.

Hyperopia can be classified in mild hyperopia (>0.5 D to ≤ +2.0 D), moderate hyperopia (>+2.0 D to ≤ +5.0 D) and high hyperopia (>+ 5.0 D) [16]. In Shandong in China, the prevalence of mild hyperopia decreases continuously from 77.4% at the age of 4 years to 7.5% at 18 years and the prevalence of moderate and high hyperopia diminishes from 14.8% at 4 years to 1.4% at 12 years and is relatively stable thereafter [14]. There is a general trend towards regression of hyperopia in China with higher regression rates in children with high hyperopia compared to children with moderate hyperopia [15]. In Germany, the prevalence of all hyperopics >+0.5 D, measured without cycloplegia, decreased from 17.2% at the age of 3 to 4.5% at the age of 11 and is relatively stable thereafter [17].

## Materials and methods

### Study design

This analysis is part of the LIFE Child study, which was established to monitor healthy child development. It consists of three cohorts, which are interrelated: the birth cohort, health cohort and obesity cohort. LIFE Child is, among others, one of the biggest ongoing longitudinal and cross sectional studies in Europe, to understand a wide range of factors influencing health and growth in children [18]. It takes place in Leipzig, Germany and aims to indicate representative data [18]. Despite many efforts, the study population shows a bias towards a higher educational and socioeconomic status [18,19]. Designed as a longitudinal and cross sectional study, participants are invited once a year for continuous measurements. At each visit, a consent form is to be signed by a parent, by the child if possible and a physician or physician assistant. The study was approved by the Ethical Committee of the medical faculty of the University of Leipzig (Reg. No. 264-10-ek) and registered with the trail number NCT02550236.

Data from 1999 participants of 1411 families from the health and obesity cohort between the age of 3 and 18 years, of which were 1046 boys and 953 girls, were analysed. The data was collected between January 2014 and May 2018.

### Measurements

Autorefraction without cycloplegia was carried out with the wavefront aberrometer ZEISS i.Profiler plus (Carl Zeiss Vision GmbH, Germany), which is based on a Hartmann-Shack sensor. The refractive error was analysed at a 3mm pupil and a vertex distance of 12mm. In our study setting, it was not possible to apply cycloplegica, as the ethical commission denied the use of cycloplegia. For conducting the measurements, the light was switched off and the window blinds were closed. Only the light emitted by the computer screens illuminated the room. Three measurements of each eye were carried out for each patient, whereby the individual eyes were measured in an alternating manner. If the children were not able to concentrate over the whole study period, the measurements were discontinued after one or two trials.

In order to relax accommodation, the focus target is defocused initially (fogging). Before the aberrometry commences this defocus is reduced and the participant can see the target clearly.

After autorefraction the uncorrected visual acuity (UCVA) and the best corrected visual acuity (BCVA) were obtained using the ZEISS i.Polatest^®^ (Carl Zeiss Vision GmbH, Aalen, Germany) with the spherocylindrical combination measured with the ZEISS i.Profiler plus. For children from 3 to 6 years and older children who were not able to read, Colt Symbols were used, for the older children letters. The test distance was 6 meters with mirror.

### Statistical analysis

According to the WHO guidelines for attained growth curves, the refraction is presented as a continous function of age. This results in smoother curves than using age intervals and provides better comparableness. The GAMLSS model allows for creating such reference curves from continuous variables [1,2]. For statistical analysis, the software R, by the R foundation, with the additional package “gamlss” was used. This method has been used in former papers analysing the LIFE Child database in order to create reference curves [20,21]. While the LMS distribution is the most commonly used with this method [2] it does not allow modelling of negative values, which are present in the spherical equivalent. We, therefore, used the slightly more flexible skew exponential power type 2 distribution (SEP2) of the same package. For comparison with (Cheng), 3rd, 50^th^, and 97^th^ percentiles were calculated, but other values could be easily obtained from the fitted models [22].

Data collected was longitudinal with the first visit at different ages. Especially for the older age groups, sufficient data is only available when data from follow-up visits are taken into account. Furthermore, some of the study population are siblings and data therefore interrelated. As this problem exists for all statistical analyses within the LIFE Child study a method has been developed to generate reference intervals from unbalanced, interrelated data [23].

Of all three measurements, the spherical equivalent (SE) of the right eye was calculated (SE = sphere + ½ cylinder). The median out of the three measurements was used for reference interval calculation. If only two measurements were obtained, the more positive one was chosen for calculation. We used this procedure, as the non-cycloplegic measurement is commonly lower than the corresponding cycloplegic measurement [24], so the more positive (or less negative) result would be closer to a cycloplegic result. In case only one measurement was taken this was used for calculation. Less than three measurements appeared mainly in young children, but already 87.4% of 3-year-olds had all three measurements taken, compared to 100% of 17 year olds. In order to evaluate as many data of different children as possible, we decided not to discard the results with less than three measurements.

## Results

**Fig 1:**
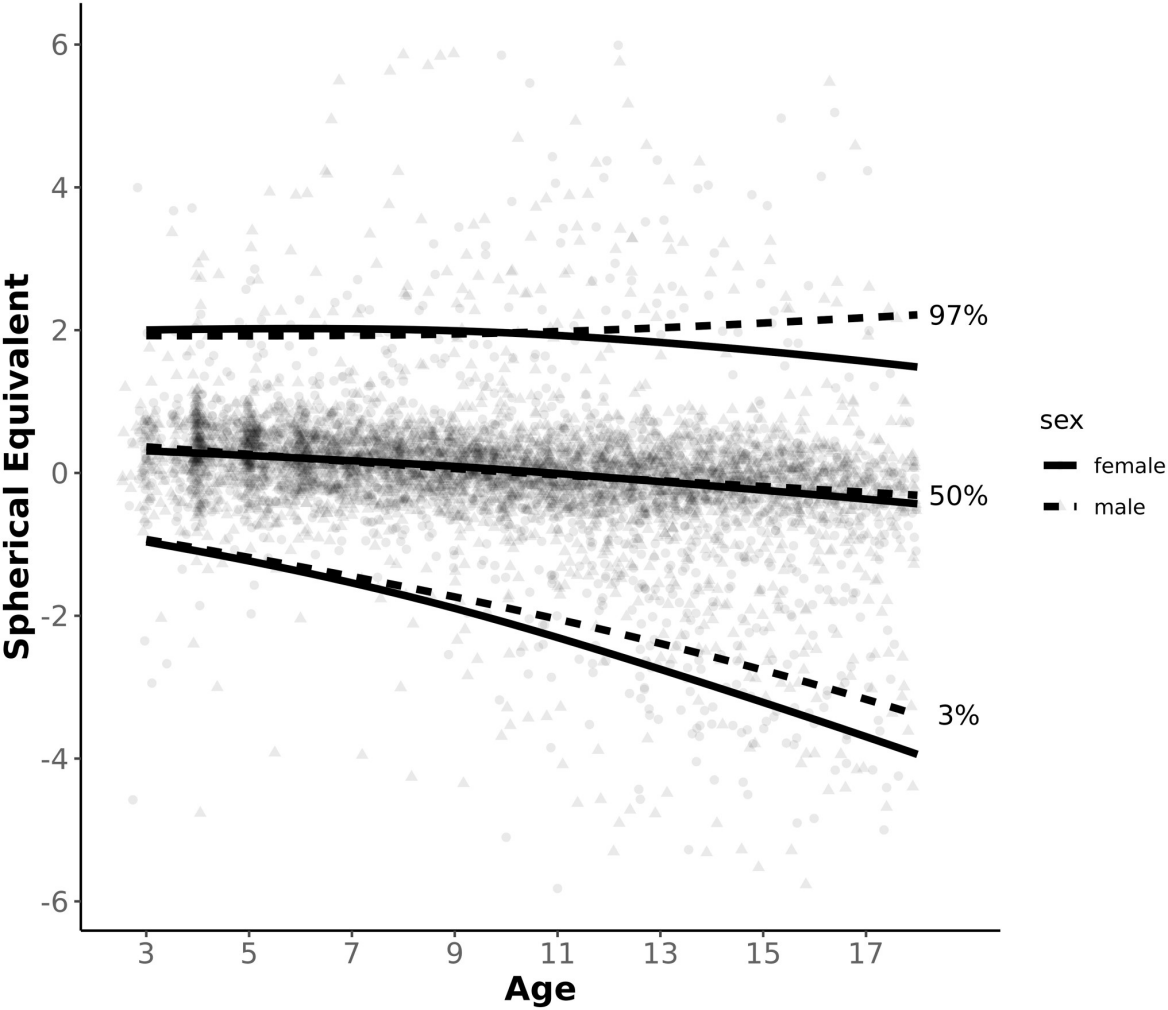
Reference curves for non-cycloplegic autorefraction (spherical equivalent) over age. Analysed were 953 girls and 1046 boys of the LIFE Child study population. The lines show the 3rd, 50th and 97th centile. Each dot represents a single measurement.

Using the GAMLSS package of the R software reference intervals were created for the refraction development over age of the right eye for boys and girls separately. Fig 1 shows the 3^rd^, 50^th^ and 97^th^ centile curve of refractive development and Table 1 shows the differences of refractive development between boys and girls. The reference intervals cover the age interval from 3 up to 18 years. The non-cycloplegic spherical equivalent, obtained by wavefront-based autorefraction is shown. We followed the most common definition of myopia for non-cycloplegic measurements and defined myopia as SE < -0.5 D (7). In the graph, the myopia cut-off is shown as dashed line.

**Table 1:** 3^rd^, 50^th^ and 97^th^ centile cut-offs in diopters for boys and girls and comparison between the genders of the LIFE study population.

Age	Boys [D]				Girls [D]				Difference (girls-boys) [D]		
	N°	C3	C50	C97	N°	C3	C50	C97	C3	C50	C97
3	61	-0.93	0.37	1.92	60	-0.96	0.31	2.00	-0.03	-0.06	0.08
4	183	-1.06	0.31	1.92	149	-1.09	0.28	2.01	-0.04	-0.04	0.09
5	170	-1.18	0.26	1.92	164	-1.23	0.24	2.02	-0.05	-0.02	0.10
6	159	-1.31	0.21	1.92	166	-1.38	0.21	2.02	-0.07	0.00	0.10
7	158	-1.45	0.16	1.93	167	-1.54	0.17	2.02	-0.09	0.01	0.09
8	172	-1.59	0.11	1.94	161	-1.71	0.13	2.01	-0.12	0.02	0.07
9	200	-1.74	0.06	1.95	151	-1.90	0.09	1.99	-0.16	0.03	0.04
10	190	-1.89	0.02	1.96	145	-2.10	0.04	1.96	-0.21	0.03	0.00
11	184	-2.05	-0.03	1.98	137	-2.31	-0.01	1.93	-0.26	0.02	-0.05
12	186	-2.21	-0.07	2.00	162	-2.52	-0.06	1.88	-0.31	0.01	-0.12
13	166	-2.39	-0.11	2.03	167	-2.75	-0.12	1.83	-0.36	-0.01	-0.21
14	160	-2.57	-0.15	2.07	163	-2.98	-0.18	1.77	-0.41	-0.03	-0.30
15	136	-2.76	-0.19	2.10	126	-3.21	-0.24	1.71	-0.45	-0.05	-0.39
16	119	-2.96	-0.23	2.14	116	-3.45	-0.30	1.64	-0.49	-0.07	-0.50
17	90	-3.17	-0.27	2.18	85	-3.69	-0.37	1.56	-0.52	-0.10	-0.61
18	35	-3.39	-0.31	2.21	27	-3.94	-0.43	1.48	-0.55	-0.12	-0.73

N° states the number of participants in each age group. One participant can be present in several age groups, but in the statistical analysis each participant is weighted equally regardless of the number of visits.

The 50^th^ centile does not differ between boys and girls and shows a general trend towards a myopic shift. At 3 years of age, the median refraction is 0.37 D for boys and 0.31 D for girls. At 18 years, the median has shifted to -0.31 D in boys and -0.43 in girls. Thus the myopic shift of the median between the age of 3 and 18 years is -0.68 D and -0.74 D for boys and girls, respectively. For both genders, the median becomes less than zero at the age of 10 years but does not become myopic (less than -0.5 D) up to the age of 18.

At 3 years of age, data does not show differences between the genders. The 97^th^ centile is 1.92 D for boys and 2.0 D for girls and the 3^rd^ centile is -0.93 for boys and -0.96 for girls. While in the hyperopic boys of the 97^th^ centile there is a subtle hyperopic shift to 2.21 D (+ 0.29 D between the age of 3 and 18), girls show the opposite trend to 1.48 D (-0.52 D in the observed period).

During the whole observation period, the gap between the 3^rd^ centile between boys and girls increases. The 3^rd^ centile cut-off is -3.39 D for boys and -3.94 for girls at the age of 18. Both genders show a myopic shift in the 3^rd^ centile, which was -2.46 for boys and -2.98 for girls.

It can be seen that the upper centiles show in general less changes throughout age groups compared to the lower centiles. With increasing age, the myopic shift of the 3^rd^ centile is faster up to the end of the observed period.

In general, there is a trend to more myopic development in girls, compared to boys.

**Fig 2:**
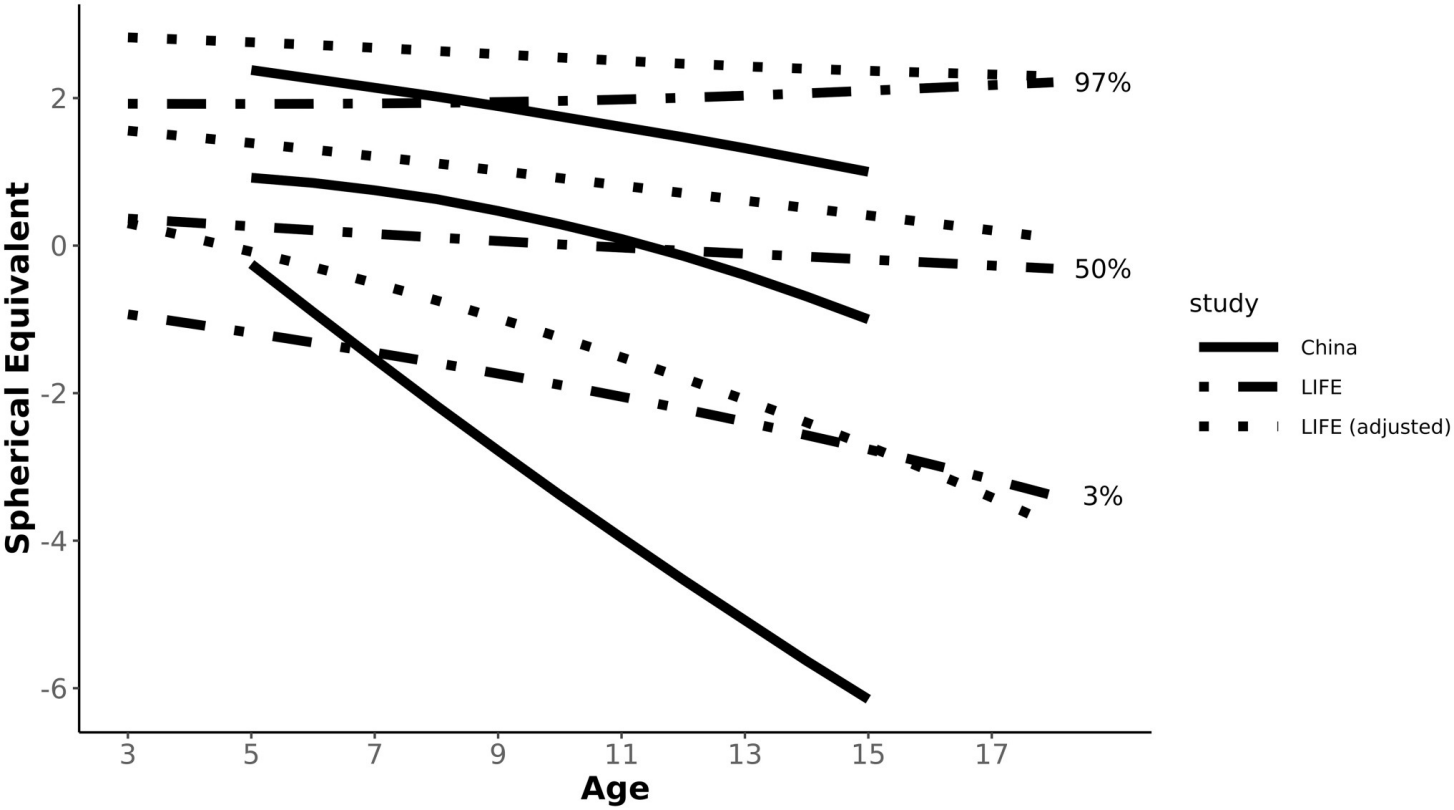
3^rd^, 50^th^, 97^th^ centile reference curves of refraction over age for boys. The dashed lines represent the data of the LIFE Child study, the dotted lines the transferred data of LIFE Child study by the calculation of Sakaridurg et al. into comparable data for cycloplegia and the continous lines show the results of the RESC study from a city in China [3].

**Fig 3:**
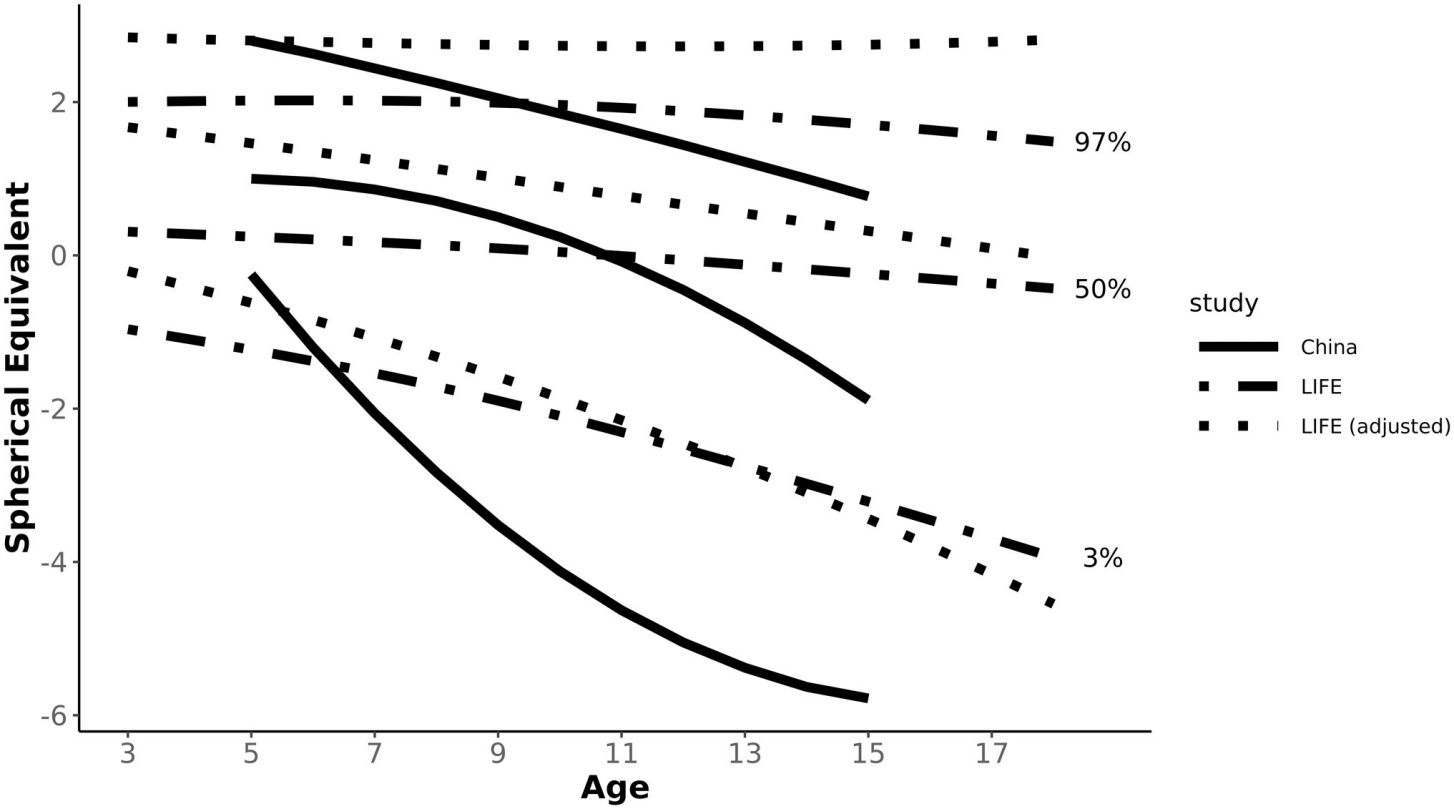
3^rd^, 50^th^, 97^th^ centile reference curves of refraction over age for girls. The dashed lines represent the data of the LIFE Child study, the dotted lines the transferred data of LIFE Child study by the calculation of Sakaridurg et al. into comparable data for cycloplegia and the continous lines show the results of the RESC study from a city in China [3].

In order to compare refraction reference curves between Germany and China we had to transfer our data from non-cycloplegic measurements into adequate cycloplegic measurement results. Figs 2 and 3 and Table 2 show reference curves for boys and girls in the RESC study in China [3] in comparison to data from the LIFE Child study in Germany.

**Table 2:** Differences of 3^rd^, 50^th^ and 97^th^ centile cutoffs in diopters for boys and girls between LIFE child data (adjusted by the calculation of Sankaridurg et al) and RECS study.

Age	Boys (Adjusted LIFE Data—RECS study) [D]			Girls (Adjusted LIFE Data—RECS study) [D]		
	C3	C50	C97	C3	C50	C97
5	0.17	0.47	0.38	-0.37	0.46	0.00
6	0.61	0.45	0.46	0.36	0.39	0.16
7	1.03	0.46	0.54	0.99	0.38	0.33
8	1.43	0.48	0.62	1.51	0.42	0.51
9	1.79	0.55	0.70	1.94	0.51	0.70
10	2.14	0.63	0.80	2.26	0.65	0.88
11	2.45	0.72	0.90	2.48	0.86	1.08
12	2.73	0.85	1.00	2.60	1.11	1.29
13	2.99	1.01	1.11	2.61	1.43	1.51
14	3.23	1.20	1.24	2.53	1.79	1.74
15	3.43	1.41	1.37	2.34	2.21	1.98

In the RESC study data was only collected from the age of 5 to 15. Therefore we can only compare refraction curves of this age group. While our data does not show differences between boys and girls in the 50^th^ centile over all age groups, the 50^th^ centile of boys and girls in China developed similarly up to the age of 10 years. Above the age of 10, there was an increasing gap between the myopic shift between the genders in China. At 15 years, girls in the 50^th^ centile were 0.89 D more myopic than boys, which is clinically relevant. In the 3^rd^ centile girls in Germany became increasingly more myopic than boys. This trend could be seen in China as well, but only up to the age of 10 years. The trend reversed, and at 15 years, boys in the RESC study were more myopic than girls.

Comparing the adjusted data for non-cycloplegic measurements, mean refraction at the age of 5 was 1.39 D in Germany and 0.92 D in China for boys and 1.46 D for German girls compared to 1.00 D for Chinese girls. The mean refraction for both genders is almost half a dioptre more myopic in China at the age of 5 years. This difference increases up to the age of 15 to 1.41 D in boys and 2.21 D in girls, showing a stronger trend towards myopia in China.

At age 5 there is little difference between both ethnicities for the 3^rd^ centile. Boys in China are slightly more myopic (0.17D) and Chinese girls are even 0.37 D less myopic than German girls. Already in 6 year old children differences begin to increase with Chinese children becoming more myopic than German children. At the age of 15, the gap is 3.43 D for boys and 2.34 D for girls.

For the 97^th^ centile, however, data at age 5 shows no differences for girls and boys are 0.38 D less hyperopic in China. For 15 year old children, the differences between the ethnicities are similar to the differences of the 50^th^ centile.

While at age 5 there is in general little difference, the Chinese population shows for all centiles a more myopic/ less hyperopic development up to the age of 15.

## Discussion

Because our data was measured without cycloplegia but Chen et al. measured refraction under cycloplegia, the data cannot be compared directly.

Sankaridurg et al. have tried to tackle this problem and proposed a calculation which allows prediction of cycloplegic refraction from non-cycloplegic refraction. In their setting, they analysed data of 4 to 15 year old children in Shanghai with and without cycloplegia. Autorefraction was carried out with the Topcon K R-8900, which is not wavefront-based. They found that the difference between non-cycloplegic and cycloplegic autorefraction was -0.63 D on average. The difference was dependent on age, refraction and visual acuity. The deviation between cycloplegic and non-cycloplegic measurements would be highest for young hyperopic children and lowest for older myopic children [25]. Accommodation is generally higher in younger children [25] and, naturally, more hyperopic children are used to accommodate even more.

For 13 year old children, Sanfilippo et al. found similar differences from non-cycloplegic to cycloplegic autorefraction with the Humphrey-598 automated refractor in Australia, suggesting that this relation can be adopted to other ethnicities [26]. Other studies in Australia using the Canon-RK-F1 autorefractor and in China with the Nikon Retinomax K-Plus autorefractor found even higher differences between cycloplegic and non-cycloplegic autorefraction [24,27].

One might argue that the calculation by Sankaridurg et al. is based on autorefraction, but not wavefront-based autorefraction. However, it has been shown that there is no difference between the two methods of obtaining autorefraction. For adults the results taken by the i.Profiler Plus (Carl Zeiss Vision, Aalen) are comparable to the results by the Canon RK 2F autorefractor [28]. The same relation has been shown for other autorefractors and Hartmann-Shak sensors [29].

Using the formula of the “model B” from Sankaridurg et al. [25] we aim to convert our set of data into the equivalent of cycloplegic data in order to be able to compare centile curves. Both Sankaridurg et al. and Chen et al. used 1% cyclopentolate instilled 5 minutes apart for cycloplegia. As both used the same cycloplegic drug and the same procedure we could compare our transferred results to the results of the RESC study. Figs 2 and 3 show the 3^rd^, 50^th^ e and 97^th^ centile for boys and girls of the RESC study (solid lines) and LIFE Child study original dataset and the transformed data to cycloplegia (dashed and dotted lines, respectively).

Through this transformation our data became more positive in general. The difference between non-cycloplegic and cycloplegic refraction decreased with age. The calculated cycloplegic comparative value was dependent on age, refraction and uncorrected visual acuity. On average, the difference between the LIFE Child measured data and the calculated values was -0.75 D. That means, that the corresponding cycloplegic data should be +0.75 D more hyperopic on average.

Chen et al. were the first study group (RESC study) to publish centile curves for refraction development in children and adolescents. Data for their study was collected in Guangzhou city in China [3]. The prevalence of myopia is much higher in Asia compared to Europe [7]. We seek to discuss the differences in percentile curves between China and Germany. Our aim is to find the point of time when overall development of refraction diverges, as the prevalence of myopia and the prevalence of pathologic myopia below -6.0 D are much higher in Asia [7].

Our study population shows overall a more hyperopic and less myopic structure. This difference increases with age. At the end of the observed age period. the 50^th^ and 97^th^ centile differences between the girls are more pronounced than differences in boys. For both genders, the largest differences can be seen in the 3^rd^ centile, which are more distinct in boys than in girls. For myopic children the difference is much higher than for the rest of the study population. While the higher prevalence of myopia and especially high myopia explains the huge deviation of the 3^rd^ centile at age 15 between both ethnicities, it is interesting that there is also a marked deviation of the 97^th^ centile. The prevalence of hyperopia of more than 2 Diopters in China in 5 year old children is 17.0% and in 15 year old children below 1% [30]. Data for Europe measured in a comparable manner was only available for the age group 25 to 29. The prevalence of hyperopia of at least one Diopter was 6.4% and high hyperopia with at least 3 Diopters 1.1% [31]. Assuming that the prevalence of hyperopia does not increase in early adulthood, the prevalence of hyperopia is very likely higher in Europe compared to Germany. While the 97^th^ centile for the adjusted German data is within the range of hyperopia for all age groups, in the 97^th^ centile of the Chinese population it is clearly visible that less than 3% of the Children at age 15 are hyperopic with more than 2 Diopters. As there was no 99.5^th^ centile given from the RECS study we were not able to compare the development of refraction of these children who are likely to stay hyperopic in both ethnicities.

The deviation of the 3^rd^ centile may be due to differences in the school system. At 5 years of age, Chinese children start pre-primary school. At 6 years, both children in Germany and China start primary school. Where there are only 5 school-days per week in Germany with lesson times from around 8:00 am to 12:00 am for the first school years and only little homework, in china school times are from around 7:30 am to 4:00 pm with more homework and less holidays [32]. As postulated before [6–8,33], this suggests that more near-work and less time spent outside is one of the reasons for the prevalence of myopia rocketing in China. But it is likely, that there is also a genetic component, as there are already differences at the age of 5 before school starts in either of the countries.

Kim and Lim have also published centile curves of a large population of the KNHANES IV-V study in Korea aged 5 to 20 years [34]. Their data was measured with the KR-8800 Topcon Autorefractor without cycloplegia. However, data in this study was not analysed separately between boys and girls. Data are only given for the 10^th^ and the 90^th^ centile. At 5 years the 90^th^ centile was +0.72 D in Korea, +1.11 D for German girls and +1.07 D for German boys. The 50^th^ centile was 0.04 D in Korea, 0.24 D in German girls and 0.26 D in German boys and the 10^th^ centile -0.75 D for Korea and -0.45 D and -0.42 D for German girls and boys, respectively. Compared to data of the LIFE Child study values for boys at 5 years do not differ much between the ethnicities with a general tendency of a more myopic structure in Korea. At 20 years however, the 90^th^ centile in Korea is -0.25 D, the 50^th^ centile -2.88 D and the 10^th^ centile -5.98 D. For German girls and boys, respectively the 90^th^ centile is 0.44 D and 0.87 D, the 50^th^ centile -0.43 D and -0.31 D and the 10^th^ centile -2.19 D and -1.82 D for 18 year olds. The data for 18 years is not given in the paper by Kim and Lim. Therefore we can only compare data at age 18 from our setting to data at age 20 in Korea. However, there is over all a more myopic setting in Korea compared to the Life Child data at 18 years. The differences are very obvious and would also be marked, if children at exactly the same age were compared. The differences are higher for the lower centiles, which show the more myopic children.

Interestingly both, the RESC study and the KNHANES IV-V study showed only little differences in the 5 year old children compared to LIFE Child data. During the whole observation period, this difference increased, leading to a more myopic setting at the study end point in China and Korea compared to Germany. For all populations, the progression rates towards myopia were higher for the more myopic children.

## Limitations

When comparing the three individual results of non-cycloplegic autorefraction in our setting the repeatability was ±0.78 D. As the fluctuation between the individual measurements was higher than the yearly progression rate of myopia, it was not possible to analyse longitudinal data, which we collected in some of the patients over up to 4 years.

Without cycloplegia, accommodation cannot be controlled and despite the fogging process during the measurement there can be some accommodation leading to results which are more myopic or less hyperopic than a comparable measurement under cycloplegia [25,35].

However, for a screening or paediatric setting cycloplegia is not feasible. Therefore doctors should be aware of the overestimation of myopia and underestimation of hyperopia depending on age and refraction. Using autorefraction as screening tool only, not for diagnosis or correction with glasses, this method still gives a good evaluation of refractive status and general outliners.

## Conclusion

For the first time, age-specific detailed refraction percentile curves of children and adolescents in Germany are presented. Compared to data from Asia there is only little difference until the age of 5 years. Thereafter, especially the difference in the 3^rd^ percentiles between the LIFE Child data and the data from Guangzhou increases dramatically. While there is only little alteration in the 97^th^ centile in Germany, the trend towards less hyperopia or myopia can be seen in China also in the upper centile. However, for both populations the myopia progression rates increase with higher baseline myopia.

In order to predict future refractive development from the current refraction, longitudinal data needs to be collected and the predictive value of our percentile curves needs to be defined.
